# CNV analysis of Meishan pig by next-generation sequencing and effects of *AHR* gene CNV on pig reproductive traits

**DOI:** 10.1186/s40104-020-00442-5

**Published:** 2020-04-21

**Authors:** Xianrui Zheng, Pengju Zhao, Kaijie Yang, Chao Ning, Haifei Wang, Lei Zhou, Jianfeng Liu

**Affiliations:** 1 National Engineering Laboratory for Animal Breeding, Key Laboratory of Animal Genetics, Breeding and Reproduction, Ministry of Agriculture, College of Animal Science and Technology, China Agricultural University, Beijing, 100193 China; 2 Department of Animal Genetics, Breeding and Reproduction and Molecular Design, College of Animal Science and Technology, Yangzhou University, Yangzhou, 225009 China

**Keywords:** *AHR*, Copy number variation, Meishan, Next-generation sequencing, Pig, Reproduction

## Abstract

**Background:**

Reproductive performance of livestock is an economically important aspect of global food production. The Chinese Meishan pig is a prolific breed, with an average of three to five more piglets per litter than European breeds; however, the genetic basis for this difference is not well understood.

**Results:**

In this study, we investigated copy number variations (CNVs) of 32 Meishan pigs and 29 Duroc pigs by next-generation sequencing. A genome-wide analysis of 61 pigs revealed 12,668 copy number variable regions (CNVRs) that were further divided into three categories based on copy number (CN) of the whole population, i.e., gain (*n* = 7,638), and loss (*n* = 5,030) CNVRs. We then compared Meishan and Duroc pigs and identified 17.17 Mb of 6,387 CNVRs that only existing in Meishan pigs CNVRs that overlapped the reproduction-related gene encoding the aryl hydrocarbon receptor (*AHR*) gene. We found that normal *AHR* CN was more frequent than CN loss in four different pig breeds. An association analysis showed that *AHR* CN had a positive effect on litter size (*P* < 0.05) and that a higher CN was associated with higher total number born (*P* < 0.05), number born alive (*P* < 0.05), number of weaned piglets, and birth weight.

**Conclusions:**

The present study provides comprehensive CNVRs for Meishan and Duroc pigs through large-scale population resequencing. Our results provide a supplement for the high-resolution map of copy number variation in the porcine genome and valuable information for the investigation of genomic structural variation underlying traits of interest in pig. In addition, the association results provide evidence for *AHR* as a candidate gene associated with reproductive traits that can be used as a genetic marker in pig breeding programs.

## Background

Sow reproductive performance is an important factor for the profitability of pig production [1]. Litter traits mainly include total number of piglets born (TNB), number of piglets born alive (NBA), number of weaned piglets (NWP), birth weight (BW), gestation length (GL), and number of stillborn piglets. Genomic variation such as single nucleotide polymorphisms can affect reproductive traits by controlling gene expression levels [2]. Copy number variation (CNV) is defined as a variable copy number of DNA segments ranging from 50 bp to several megabases (Mb) compared with a reference genome [3]. CNVs are useful molecular markers that influence gene expression and phenotype through various mechanisms such as gene dosage modification, gene structure disturbance, and loss of regulatory elements or polymorphisms [4].

The functional relevance of CNVs to genetic diseases [5], immunity [6], and reproduction has been investigated in several studies [7, 8], which have revealed new markers for complex traits in humans and important economics traits in domestic animals. In humans, several studies of CNV have shown that it is associated with susceptibility to Mendelian diseases and complex genetic diseases such as cancer [9] and various congenital defects [10]. In cattle, CNV of the *PRAMEY* gene was found to be associated with testis size and bull fertility in Holstein [7], and a deletion-type CNV encompassing *ANXA10* was shown to be critical for embryonic development in Japanese Black cattle [8]. In pigs, previous studies used array-based comparative genomic hybridization (aCGH), SNP-array or next-generation sequencing methods to detect CNVs. For instance, Chen et al. identified 1,315 putative CNVs belonging to 565 CNVRs in 1,693 pigs from 18 diverse populations using Porcine SNP60 BeadChip and PennCNV algorithm and revealed 7 copy number variable genes as candidate genes related to carcass length, backfat thickness, abdominal fat weight, length of scapular [11]. Revilla et al. [12] identified 1,279 CNVs and 540 CNVRs using whole genome data and they provided candidate genes for fatty acid composition and growth traits. Jiang et al. [13] indicated the total CNVRs amounted to 4.0% based on the porcine genome (*Sus scrofa* build10.2) and most CNVRs fell into the interval between 10 kb and 20 kb. Paudel et al. identified 1,408 regions, comprising 17.83 Mb of the porcine genome (*Sus scrofa* build10.2). Many of the identified CNVRs are relatively small, the size of CNVRs ranges from 6 to 98 kb, 78% of the CNVRs that were identified is between 6 and 15 kb. CNVRs covered 0.7% of the porcine genome [14]. Although the size ranges and coverage of CNVR detected in previous swine studies were different, the functional genes were consistent, such as olfactory receptor, which is known to play a prominent role in food foraging and mate recognition in Sus. Keel et al. [15] using three methods to identify CNVRs covered 0.94% of the porcine genome and the number of CNVRs per animal ranged from 0 to 348, with a mean of 157.8. However, the CNVRs were most overlapped with reproductive traits [15]. A limitation in most of the aforementioned CNV studies in swine is using the Sscrofa 10.2 genome builds.

Candidate CNVs and genes associated with complex traits have also been reported [11, 16, 17]. A CNV of the *MSRB3* gene encoding methionine sulfoxide reductase has been shown to increase porcine ear size [18]. CNV of the *MTHFSD* gene affects litter size in the Chinese indigenous Xiang pig [19]. Meanwhile, *MCHR1*, *PPARα*, *SLC5A1*, and *SLC5A4* CNVs have been implicated in fat-related functions [16]. Meishan is a Chinese swine breed that it is well known for its high prolificacy; however, the genetic basis for this high fecundity is largely unknown. In addition, the using of next-generation sequencing (NGS) in this study allows to identify a wide range of CNV, especially many small CNVs that are missed when using SNP chips.

To address these issues, in this study we performed a genome-wide CNV analysis in Meishan pigs by next-generation sequencing (NGS). We also performed a comparative analysis with Duroc pigs to identify the putative CNV regions (CNVRs) only existed in Meishan as well as an association study between genes in CNVRs and pig reproductive traits.

## Methods

### Animal ethics

Animal care and next-generation sequencing were approved by the Institutional Animal Care and Use Committee of China Agricultural University (Beijing, People’s Republic of China; permit No. DK1023).

### Sample collection

A total of 61 pigs were analyzed by NGS including 32 Meishan pigs from Kunshan City and 29 Duroc pigs from Yancheng City of Jiangsu Province. For qPCR analysis, we obtained 853 blood samples from Beijing Liuma Technology Co. (Beijing, China) and Meishan Pig Conservation Breeding Co. (Kunshan, China). The samples were from four pig breeds: Duroc (*n* = 171), Landrace (*n* = 176), Yorkshire (*n* = 478), and Meishan (*n* = 28). Reproductive data such as TNB and NBA were available for all sampled individuals.

Genomic DNA was isolated from pig blood samples using the Genomic DNA Extraction Kit Qiagen DNeasy Tissue kit (Qiagen, Hilden, Germany) according to the manufacturer’s instructions. DNA quality was verified with a NanoDrop spectrophotometer (Thermo Fisher Scientific, Waltham, MA, USA) and by 0.8% agarose gel electrophoresis.

### Re-sequencing and CNV detection

Next-generation sequencing library preparation involves generating a collection of DNA fragments for sequencing. In the library preparation, 50 ng genomic DNA was fragmented in 16 μL of TE. After fragmentation, samples were end-repaired using the New England Biolabs (NEB) sample preparation kit and protocol (NEB Next DNA Sample prep, E6000S), with incubation time of 30 min. Following end-repair, a single dA was added to the end of each Blunted. After A-tailing, DNA ligation was performed. Once ligation had been assessed, the adapter ligated library was PCR amplified. Finally, the libraries generated for 61 pigs were sequenced on an Illumina HiSeq2000 platform at Novogene (Beijing, China). All paired-end reads reached the length of 125 bp, with an average insert size of 460–490 bp and the standard deviation of 11–14 bp estimated for all samples.

In the preprocessing of CNV detection, all the data were removed adapters and low quality reads (the quality score lower than 20) using NGSQC Toolkit [20]. The filtered reads were further aligned to pig reference genome (Sus sacrofa 11.1) by Burrows-Wheeler Aligner (BWA) with the default parameter. The average of read mapping ratio was 96% for 61 pig samples.

The CNVs were detected using CNVnator (v0.3.3) software [21] and CNVcaller (RRID:SRC 015752) [22]. The CNVnator captured the read-depth signal in genomic regions with different CNs and genotyped both deletions and duplications with the correction for GC bias. For each pig, the aligned files were processed to identify genome-wide CNVs (except those on the X and Y chromosome) with standard parameters and 200-bp bins [23]. The CNVcaller applies robust signal detection and noise deduction methods on basis of RD algorithm to increase the computational efficiency in complex genomes. We ran the CNVcaller by population levels for Meishan and Duroc breeds with the default arguments [22].

After the CNV detection, all CNVs of each individual detected by CNVnator and CNVcaller were merged by one-to-one correspondence when the overlap is of at least 1 bp by bedtools [24]. Then we merged the CNVs of Meishan and Duroc into CNVRs by breed. For each breed, the CNVRs were defined as the CNVs identified in three or more individuals when the overlap is of at least 1 bp.

For CNVs that overlapped among different individuals, CNVRuler software [25] (http://www.ircgp.com/CNVRuler/?ckattempt=1) was used to define two types of common CNVRs, loss and gain, along with fragmented CNVs. CNVRs were used to construct a CNV map for Meishan and Duroc pigs, and fragmented CNVs were used for subsequent CNV comparisons in all pigs.

To detect CNVs between Meishan and Duroc populations, we used the relative frequency difference (RFD value) [26] to assess CNV diversity within each breed based on fragmented CNV frequency. Fragmented CNV means that a single large CNV is fragmented into multiple smaller calls [27], which is mainly used in CNV comparison among different populations. The RFD of the Meishan population relative to the Duroc population was calculated as follows: RFD_Meishan-Duroc_ = (F_Meishan_ − F_Duroc_)/F_Meishan-Duroc_, where F_Meishan_, F_Duroc_, and F_Meishan-Duroc_ represent the fragmented CNV frequency in the Meishan population (with CNV discarded for both F_Meishan_ and F_Duroc_ < 0.05). We calculated both deletion and duplication of RFD values for mixed CNVs in all pigs of both breeds.

### GO enrichment analysis and functional classification

KOBAS 3.0 is a web server for gene/protein functional annotation (Annotate module) and functional gene set enrichment (Enrichment module). Thus, to provide insight into the functional enrichment of the CNVRs, we performed gene ontology (GO) and Kyoto Encyclopedia of Genes and Genomes (KEGG) pathway analyses for the genes in CNVRs using KOBAS 3.0 (http://kobas.cbi.pku.edu.cn/kobas3/?t=1) and PANTHER 15.0 classification system (http://www.pantherdb.org).

### CNV type assay

We evaluated CNV of the *AHR* gene using qPCR and 2^−ΔΔCT^ method. Primers used for qPCR were designed using Primer-BLAST (http://www.ncbi.nlm.nih.gov/tools/primer-blast). We selected one segment of the *GCG* gene as the reference locus since this gene is highly conserved across species and is present as a single copy in animals [28]. Primer sequences for *AHR* and *GCG* are shown in Table 1. To ensure comparability between *AHR* and *GCG*, we first determined the amplification efficiency of each assay using serial dilutions of 100 ng DNA prepared in triplicate. The threshold amplification efficiency of primers used in this study was 1.99–2.01.

**Table 1:** Primer sequences for transcripts used in real-time quantitative PCR

Gene	Primer sequence (5′→3′)	Annealing temperature, °C	Product length, bp
*AHR*	Forward: ACTACCACCCATCTTCACCCG	60	183
	Reverse: CAACACACATCAATGCTTCCC		
*GCG*	Forward: GAATCAACACCATCGGTCAAAT	60	147
	Reverse: CTCCACCCATAGAATGCCCAGT		

CNVs in 853 samples were detected by qPCR on a LightCycler 480 Real-Time PCR System (Roche, Basel, Switzerland) using DNA according to the manufacturer’s protocol. PCR amplifications were performed in a total volume of 20 μL consisting of the following reagents: 1 μL DNA (around 50 ng), 1 μL (20 pmol/μL) of both forward primer and reverse primer, 10 μL of Master Mix (2×) and water (Roche Applied Science). PCRs were run as follows: 5 min at 95 °C followed by 40 cycles at 95 °C for 10 s and 60 °C for 10 s. All PCRs were performed in 96-well clear reaction plates (Roche Applied Science). Relative expression levels were estimated with the cycle threshold (2^−ΔΔCt^) method [29], which compares the ΔCt value (Ct of the target − Ct of the control region) of samples with CNV to that of the calibrator sample. The CN of the *AHR* gene was confirmed based on the assumption that there were two copies of the DNA segment in calibrator animals. The CNV type of the *AHR* gene was defined as loss (fewer than two gene copies) and normal (two gene copies as in the positive control) according to previous studies [30, 31]. The qPCR assays for each individual were performed in triplicate.

### Association analysis

To determine the effect of CNV on pig reproductive traits, we performed an association analysis using SAS v.9.2 software (SAS Institute, Cary, NC, USA) according to the model:

*y*_*ijklmn*_ = *μ* + *H*_*i*_ + *Y*_*j*_ + *S*_*k*_ + *P*_*l*_ + *CNV*_*m*_ + *e*_*ijklmn*_*.*

where *y*_*ijklmn*_ is the phenotypic value of each trait in pigs; μ is overall population mean; *H*_*i*_, *Y*_*j*_, and *S*_*k*_ are fixed effects of farm (two farms), year (3 years), and season (four seasons), respectively; *P*_*l*_ is parity; *CNV*_*m*_ is genotype effect; and *e*_*ijklmn*_ is the random residual with e~N (0, Iσ_e_^2^) (where I is a diagonal matrix and σ_e_^2^ is the residual error variance). A *P* value < 0.05 was considered statistically significant for each test. Five reproductive traits including TNB, NBA, NWP, BW, and GL were examined in the association study of Landrace (*n* = 176) and Yorkshire (*n* = 478) pigs. We used the false discovery rate (FDR) test for significance threshold. And 5% FDR as guideline to control overall false positive during multiple testing.

## Results

### Sequencing and CNV detection

To detect genome-wide CNVs in Meishan and Duroc pigs, we performed whole-genome resequencing of 32 unrelated Meishan and 29 Duroc pigs. The mapped read depth ranged from 6.08 to 10.96, with an average depth per sample of 8.20, which was calculated using SAMtools [32] software (Table S1).

**Fig. 1:**
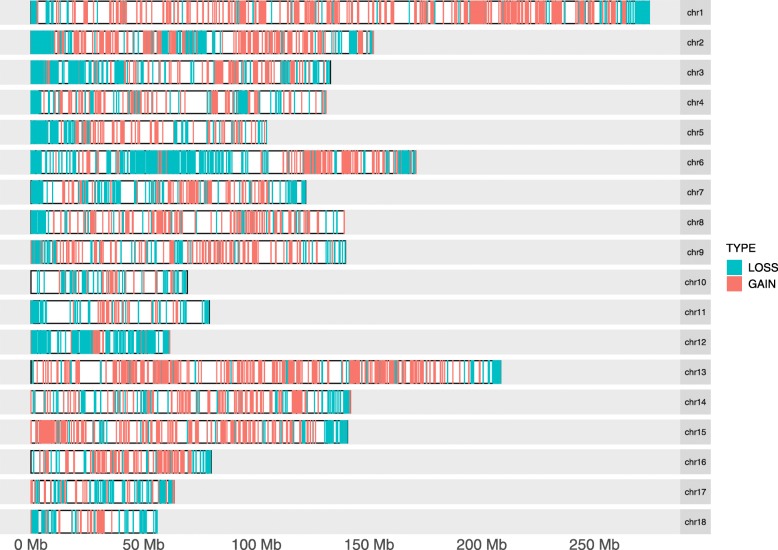
The overall CNVR maps for Meishan and Duroc pigs in the 18 autosomal chromosomes. Two types of CNVR were identified including gain (red), and loss (light blue)

**Fig. 2:**
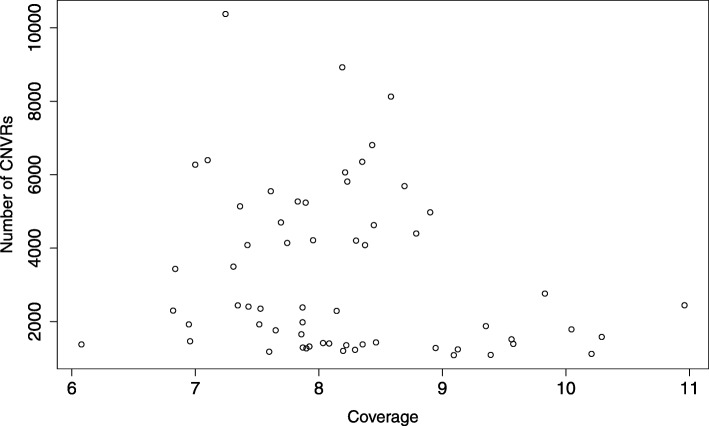
Link between the number of variants and the sequence coverage for each animal

We identified 8,282 CNVRs in the Meishan pigs, including 3,724 deletions and 4,558 duplications (Tables S2–5). And we identified 6,700 CNVRs in the Duroc pigs, consisting of 2,029 deletions, 4,670 duplications and 1 mixed (Tables S2, S3 and S6). Among them, the median number of duplications was 1,999 and the median number of deletions was 1,999. These CNVs are located in all 18 autosomal chromosomes with a mean size of 3,721.53 bp ranging from 199 bp to 279,799 bp of Meishan pig (Table 2). On average, we identified 258 and 231 variants each animal of Meishan and Duroc pigs, respectively. The CNVRs covered 1.10% and 0.99% of the porcine genome (Sscrofa 11.1) in Meishan and Duroc pigs, respectively (Table 2). All the CNVR maps for Meishan and Duroc pigs were showed in Fig. 1. In addition, we also calculated the relationship between sequence coverage and the number of CNVs identified in each individual (Fig. 2).

**Table 2:** Descriptive statistics of copy number variant identified for two breeds

Breeds	No. CNVR	No. losses	No. gains	CNV min, bp	CNV max, bp	CNV mean, bp	CNV median, bp	Coverage, kp	Coverage, %
Meishan	8,282	3,724	4,558	199	279,799	3,721.53	1,999	30,821.72	1.10%
Duroc	6,700	2,029	4,670	199	598,399	4,164.97	1,999	27,905.32	0.99%

### Frequency of variants across animals

**Fig. 3:**
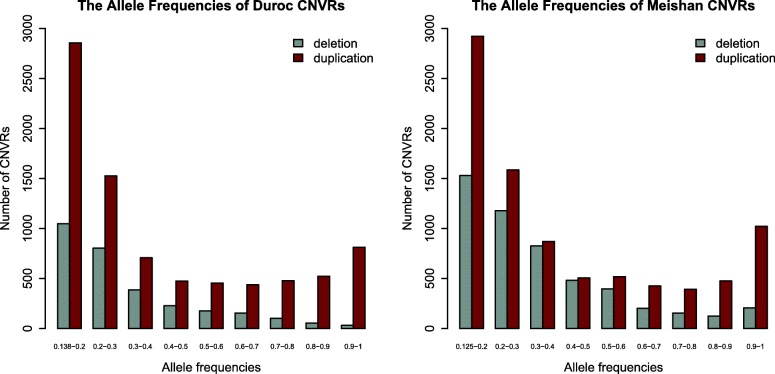
The allele frequencies of variants in the Duroc and Meishan pigs (*n*= 61)

We calculated the allele frequencies of the CNVRs in the Duroc and Meishan pigs separately (Fig. 3). Results showed the detecting frequency for duplication was higher than that for deletion. For Meishan pigs, the percentage of carriers for each variant varied from 12.5% (4 animals out of 32) to 100% (32 animals out of 32) and 34.22% of the detected CNVRs were observed in 4 (frequency 12.5%) to 6.4 (allele frequency 20%) animals. Such pattern also observed in Duroc pigs (34.69% of the identified CNVRs were existed in 4 to 5.8 animals).

### Population structure analysis of Meishan and Duroc pigs

**Fig. 4:**
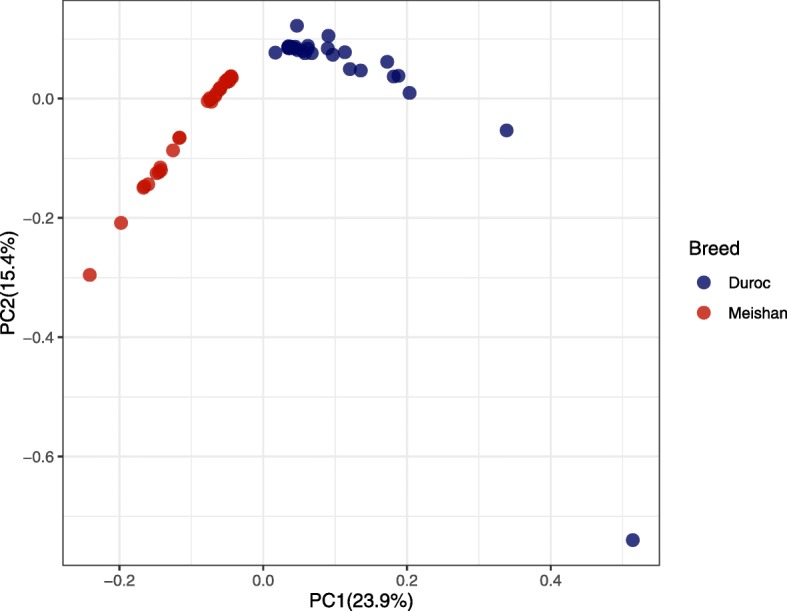
PCA plot based on the first two principal components in the Meishan and Duroc pigs. The two breeds were clustered to two groups

We analyzed the population structure of the sequenced Meishan and Duroc pigs by using Principal Components Analysis (PCA). Results showed that there was a clear distinction between Meishan and Duroc pigs based on two principal components (the variance ratio of the two major components is 23.9% and 15.4%) (Fig. 4).

### Comparison with CNVRs identified in previous reports

We compared our results of CNVRs to those identified in several previous swine studies. Results showed varying levels of overlapping CNVRs between our studies. Here, we used a stringent definition to identify overlapping CNVRs, i.e., where two CNVRs were considered overlapped when they shared at least 75% bases.

The highest percentage of overlap in CNV events identified between this work and previous studies was 4.62% (Table 3). The average overlap was 2.13%, which was lower than that in previous study (average of 4.33% overlap) [15].

**Table 3:** Comparison of CNVRs identified in this study to results from other studies (based on the Sscrofa 11.1 genome assembly)

Platform	Study	Breeds	Samples	No. of CNVRs (NO. before mapping)	No. of overlapped CNVRs from this study	Percent of overlapped CNVRs from this study
Next-generation sequencing	[12]	2	7	416 (540)	107	0.84%
Next-generation sequencing	[34]	2	16	2,265 (3,118)	262	2.07%
Next-generation sequencing	[13]	10	13	1,903 (3,131)	585	4.62%
Porcine SNP60	[11]	18	1693	243 (565)	153	1.21%
Next-generation sequencing	[14]	7	14	754 (1,408)	254	2.00%
1 M aCGH	[35]	9	12	436 (758)	180	1.42%
Next-generation sequencing	[36]	13	49	1,906 (3,131)	172	1.36%
Next-generation sequencing	[15]	3	240	3,538	449	3.54%
This Study				12,668		

Note: CNVRs were converted to Sscrofa 11.1 coordinates using the liftOver tool. Successfully mapped CNVRs are shown in the CNVRs column with the original number of published CNVRs (Sscrofa 10.2) shown in parentheses

### Gene annotation and functional analysis of the CNVRs

A total of 3,554 genes from the Ensembl annotation of the Sscrofa 11.1 genome were identified to be overlapping with our detected 12,668 CNVRs (Table S7), including 1,914 known genes and 1640 unknown genes (NA). Using PANTHER’s statistical overrepresentation test to inspect GO terms mapping to CNV-overlapped genes, we identified that CNVRs enriched for genes related to sensory perception, detection of stimulus, development, metabolic and nervous system process for biological process, which is consistent with previous studies [13]. Molecular function terms were related to G protein-coupled receptor activity, catalytic activity, sensory organ development and cation binding were significantly overrepresented in the genes overlapped by CNVR (Table S8), which were also observed by Paudel et al. [14].

KEGG analysis using KOBAS 3.0 for the total 3,514 *Sus scrofa* genes showed CNVRs are significantly enriched in Pathways related to disease and immunity (including IFN, ILR and TNF genes), reproduction, and development (Table S9, such as Pathways in cancer (corrected *P* value = 5.02E-07), Rap1 signaling pathway (corrected *P* value = 2.11E-05), Wnt signaling pathway (corrected *P* value = 0.024) as well as MAPK signaling pathway (corrected *P* value = 3.1706E-04). We found 11 genes were in the olfactory transduction pathway, however, the *P* value was more than 0.5.

Considering that the limited annotation for pig, we also performed KEGG analysis based on *Homo sapiens* (on the bottom of Table S9). We found the olfactory transduction pathway was over-represented, including 27 genes (on the bottom of Table S9).

### Comparative analysis of CNVRs in Meishan and Duroc

To determine whether the CNVs that we identified in Meishan differed from those in Duroc, we identified 6,387 CNVRs comprising 17.17 Mb in Meishan (Table S10). These CNVRs were further divided based on CN into gain (*n* = 3,348) and loss (*n* = 3,039), comprising 8.21 Mb and 8.95 Mb, respectively. The Ensembl gene annotation set (http://www.ensembl.org/) facilitated identification of a total of 6,387 CNVRs overlapping 2,610 genes.

To evaluate the contribution of CNVRs to the high prolificacy of Meishan pigs, we compared Meishan CNVRs of Meishan pigs with those of Duroc pigs and identified the regions that differed significantly between the two breeds. We extracted 17.17 Mb regions (Table S10) that was not found in the Duroc group. This region included 6,387 CNVRs overlapping 2,610 genes, of which *AHR*, *ESR2*, *STAT3* and *FSHR* are closely associated with reproductive traits. For example, the *AHR* gene has been linked to a larger litter size in European pigs [33]. These results demonstrate that Meishan pigs have given CNVRs comparing to Duroc pigs that are potentially associated with prolificacy.

We used the statistical parameter RFD [26] to detect selective sweeps in Meishan pigs based on fragmented CNV frequencies (split with CNVRuler software) between Meishan and Duroc pigs. The highest 10% absolute RFD value for each breed was used as the threshold (Meishan, 1.66; Duroc, 1.89) to identify fragmented CNVs. In total, we found 1,099 fragmented CNVs (11.7 kb on average) overlapping 443 genes. Six of the genes were closely related to reproduction, including the *AHR* gene encoding aryl hydrocarbon receptor.

### *AHR* CNV is associated with pig reproductive traits

Previous studies have employed qPCR to evaluate CNVs and their effects in Chinese bulls [37]; for instance, CNV of the *TSPY* gene was detected in 14 different cattle breeds [38]. The *AHR* gene is known to play a critical role in regulating reproductive lifespan and fertility and establishing an optimal environment for fertilization [39], as well as is important in ovarian function. *AHR* mRNA and protein expression varies according to the reproductive tissue and estrous cycle phase, suggesting their involvement in the regulation of reproductive function in female pigs [40]. Indeed, the loss of *AHR* gene expression in mutant mice and *AHR* overexpression can lead to adverse phenotypes in the female reproductive organs and impaired reproductive function [41–43]. On the basis of these observations, we speculate that *AHR* CNVs may affect reproductive performance in pigs.

To investigate the functional significance of different CNV types of the *AHR* gene in terms of pig reproductive traits, we evaluated *AHR* CN in four pig breeds (Meishan, Duroc, Landrace, and Yorkshire) by quantitative (q)PCR. The porcine *AHR* gene is located at chr9: 86,511,369 to 86,555,943 (Sscrofa 11.1) and overlapped with the CNV region: chr9: 86,518,401–86,520,000 (Table S10). We designed primer to amplify *AHR* and GCG gene. The primer pair for *AHR* started from 86,553,480 to 86,553,662 and the detection sequence size is 183 bp. The detection sequence is located in the eleventh exon of *AHR* gene. We assessed the efficiency of amplification and calculated the correlation coefficient for the target gene *AHR* and the reference glucagon gene (*GCG*); the results showed a high degree of precision in the determination of relative CN. In our study, CNVs of high quality were detected in four pig breeds through qPCR, demonstrating that this approach could be useful for other CNV studies in pigs.

The 2^−ΔΔCT^ value in all breeds ranged from 0.5–2.5 (Table S11). The pigs were divided into two classes: those with 2^−ΔΔCT^ values ranging from 0.5–1.5 as one copy (loss type) and those with values of 1.5–2.5 as two copies (normal type). Among the 853 samples analyzed, both normal and loss types were observed in Duroc, Landrace, and Yorkshire whereas normal CN was observed in Meishan. The frequency of the two types also differed across breeds: the rank order of proportion of individuals with normal CN was Duroc (46.1%) < Yorkshire (66.9%) < Landrace (67.0%) < Meishan (100%).

CNVs may affect the phenotype by altering the transcription of genes within or adjacent to a CNVR, which ultimately affects protein levels. We evaluated the association between CNV type and pig reproductive traits (i.e., TNB, NBA, NWP, BW, and GL) in Landrace and Yorkshire pigs, using a general linear model (Table 4). We found that TNB and NBA were significantly associated with the *AHR* gene CNV type in the Yorkshire breed (*P* < 0.05). Moreover, TNB and NBA were higher in individuals with the normal type compared to the loss type (*P* < 0.01); this trend was also observed in Landrace pigs, with a higher TNB in normal- than in loss-type individuals (*P* < 0.05). These findings suggest that the *AHR* gene CNV positively affects TNB and NBA in pigs.

**Table 4:** Association analysis of *AHR* CNV types with reproductive traits

Breeds	CNV type	TNB	NBA	BW	NWP	GL
Yorkshire	Loss	11.55 ± 0.28^b^	10.84 ± 0.19^b^	16.80 ± 0.27^a^	9.81 ± 0.17^a^	115.15 ± 0.10^a^
	Normal	12.00 ± 0.24^a^	11.46 ± 0.13^a^	16.90 ± 0.19^a^	9.88 ± 0.12^a^	115.02 ± 0.08^a^
	*P*-value	**0.002**	**0.0002**	0.017	0.085	0.332
Landrace	Loss	11.22 ± 0.30^b^	10.26 ± 0.29^a^	16.10 ± 0.47^a^	9.00 ± 0.29^a^	115.79 ± 0.32^a^
	Normal	11.75 ± 0.25^a^	10.72 ± 0.24^a^	16.54 ± 0.40^a^	9.21 ± 0.24^a^	115.56 ± 0.27^a^
	*P-*value	**0.039**	0.061	0.270	0.408	0.408

Note: Values with different superscripts (^a,^^b^) within the same line differ significantly at *P* < 0.05

*TNB* total number born, *NBA* number born alive, *NWP* number of weaned piglets, *BW* birth weight, *GL* gestation length

## Discussion

In this study, we detected 6,700 and 8,282 CNVRs in Duroc and Meishan pigs, respectively, accounting for approximately 0.99% and 1.10% of the reference pig genome (*Sus scrofa* 11.1). To the best of our knowledge, the present study is the first analysis of CNVRs for Meishan pigs through large-scale population resequencing. Notably, compared with Duroc pigs, Meishan pigs show an excess of CNVRs. The Meishan pig breed is well known for its high prolificacy. We identified CNV of the *AHR* gene as being potentially related to the prolificacy of Meishan pigs. In addition, qPCR analysis of the *AHR* gene in a large population of multiple pig breeds suggested that CNV of the *AHR* gene is associated with TNB and NBA. Furthermore, TNB was higher in Yorkshire and Landrace pigs with normal CN as compared to those with CN loss. These findings suggest that molecular marker-based breeding can improve pig production.

### Characteristic and functional analysis of the CNVRs

The average frequency of duplications was higher than that of deletions in both Duroc and Meishan pigs in the present study. Revilla et al. also found duplications showed a higher average frequency than did deletions (106 vs. 77) in a global analysis of CNVs in swine [12]. Similar pattern was also observed in another CNV study in porcine using a 60 k SNP BeadChip, the predicted status for the CNVRs in this study was 38.7% for gain, and 16.3% for loss [44]. This proportion may be related to natural selection, as it is assumed that the genome is more tolerant to duplications than to deletions [45]. We found the sequence coverage was not correlated with the number of identified CNVs in each animal, which is distinct from this results in cattle [46].

The concordance in this study between previous CNV studies is limited. Potential reasons for the differences between our results and these studies may be due to the difference in population size and genetic background between our study and others, different call algorithms for CNV detecting. In addition, our results were based on the Sscrofa11.1 genome assembly, while most of the previous works were based on Sscrofa 10.2. Warr et al. indicated there was dramatically difference between Sscrofa11.1 and Sscrofa 10.2 version and many problems in the10.2 version have been solved in the 11.1 version [47].

Genes located in CNV regions have a wide spectrum of molecular functions and provide a resource for investigating the biological relationship of CNVs with the genetic basis of phenotypic variations. The GO enrichment and KEGG analysis revealed that genes in CNVRs participated in G protein-coupled receptor activity, sensory organ development, and olfactory transduction, which were related to the olfactory receptors (OR). OR gene family is the most well characterized CNV-related genes in humans [48] and one of the largest gene families in porcine [49]. Besides the OR gene family, we also found some genes involved in immunity and cytochrome P450, such as CYP4A24, CYP2C42 and CYP3A29. These results were observed in previous swine CNV studies [12, 14], and together with ORs, CNV in CYP450 genes suggests a relevant role of these genes in the organism’s adaptation to rapid changes in the environment [14]. Among KEGG pathways, we found the Ras signaling pathway and MAPK signaling pathway were included. The mitogen-activated protein kinase (MAPK) pathway is known to have an important role in numerous male reproductive processes, including spermatogenesis [50], sperm maturation and activation, capacitation and acrosome reaction, before fertilization of the oocyte [51]. P38 MAPK, one of the major family of protein kinases, might be involved in FSH-induced meiotic resumption of oocytes [52]. These findings provided insight for the function of pig CNVs.

### The copy number of *AHR* gene is associated with pig’s litter traits

The difference in *AHR* CN among Meishan and three other pig breeds (Landrace, Yorkshire, and Duroc) may be attributable to their diverse genetic backgrounds. Similar findings were reported in studies of CNVs in bovine populations [53]. We found that the *AHR* CN was normal in all tested Meishan individuals and that CN loss was non-existent, unlike in the other three pig breeds. Meishan has one of the highest rates of fecundity and the highest TNB among pig breeds in China. Among the European commercial breeds Duroc, Landrace, and Yorkshire, two *AHR* gene copies were detected in 46.1, 67.0, and 66.9%, respectively, of the tested population. Previous studies indicated the Landrace and Large White were much more closely related than Duroc at the genome-wide level [54, 55], thus the higher proportion in Landrace and Yorkshire compared to Duroc could be attributed to the differences in the genome level among the three breeds. Landrace and Yorkshire were commonly used as maternal pigs, which are selected in the artificial breeding process based on large litter size. In contrast, Duroc is used as a paternal pig and is therefore selected for different traits, which may also be associated with the different proportions of *AHR* gene copies among these breeds. These observations reveal the potential role of *AHR* in influencing the phenotype of different pig breeds and we speculated that altering the *AHR* CN may increase litter size.

Numerous studies have reported that CNVs can affect the production and reproductive traits of livestock animals [37, 56]. For instance, CNVRs encompassing multiple genes associated with cattle production such as milk fat and protein yield have been reported [57], and the *MTHFSD* gene was found to be associated with litter size in Xiang pigs [19]. In the present study, an association was established between *AHR* CNV and reproductive traits such as TNB and NBA in Landrace and Yorkshire breeds, although no significant association was observed between CN and BW, or GL. AHR is a ligand-activated nuclear transcription factor that can transduce extracellular signals through DNA binding-dependent and -independent mechanisms [58]. In mammals, AHR plays an important role in primary follicle formation and regulation of follicle number [41], and can affect the follicle growth rate by regulating estradiol in mice [59]. In pigs, the *AHR* gene is known to be associated with reproductive traits [40] and litter size [33]; this was confirmed by the observation of the present study that CNV of the *AHR* gene was associated with TNB and NBA. We speculate that CNV affects the expression of the *AHR* gene in a dose-dependent manner, which in turn affects follicular proliferation and promotes ovulation, thereby increasing TNB. Taken together, our findings demonstrate that *AHR* CN is a useful marker for improving pig productivity; however, additional studies are required to confirm the molecular basis for the relationship between *AHR* gene CNV and reproductive traits.

## Conclusions

NGS-based analysis has been widely applied to identify CNVs and has led to significant progress in porcine CNV detection. We identified 12,668 CNVRs with an average size of 3.78 kb comprising 47.93 Mb of the porcine genome, which cover a small (1.71%) fraction of the pig genome. Moreover, we found the small size CNVs (<10 kb) were abundant, which accounts for almost one-half of all the CNVs. The inferred CNV regions include 3,554 genes providing an important resource for future analyses on phenotypic variation in pigs. In addition, we identified *AHR* gene, which showed associations with several of the reproductive traits. Association analysis study indicated that *AHR* CN had a positive effect on litter size and that a higher CN was associated with higher total number born, number born alive, and birth weight. We believe that our study makes a significant contribution to the literature as it provides information for the investigation of genomics structural variation underlying traits of interest in the Meishan pig, which is one of the most prolific pig breeds. Although the genetic basis for their fecundity is not well understood, molecular marker-based breeding can improve pig productivity. These findings contribute to facilitate the further identification of trait-related CNVRs.

## Supplementary information

**Additional file 1 Table S1.** Overall details of all pigs and their classification. **Table S2.** Overview of deletions for two pig breeds. **Table S3.** Overview of duplications for two pig breeds. **Table S4.** Detection of CNVRs in Meishan population. **Table S5.** Overview of CNVRs for Meishan population. **Table S6.** Detection of CNVRs in Duroc population. **Table S7.** Annotation of all the CNVRs for Meishan and Duroc pigs. **Table S8.** GO Enrichment Analysis for CNVR gene set by PANTHER. **Table S9.** KEGG and GO Enrichment Analysis for CNVR gene set by KOBAS. **Table S10.** Overview of given CNVRs only found in Meishan pigs. **Table S11.** CNV types of individuals on AHR CNV in four pig breeds.

## Data Availability

A total 61 pig samples with 1,403.35Gbases were uploaded to NCBI with BioProject ID: PRJNA378496.

## Abbreviations

AHR: Aryl hydrocarbon receptor
BW: Birth weight
CN: Copy number
CNV: Copy number variation
CNVRs: CNV regions
GL: Gestation length
NBA: Number of piglets born alive
NGS: Next-generation sequencing
NWP: Number of weaned piglets
TNB: Piglets born
